# The Medical Selection of Lives for Assurance
*The Medical Selection of Lives for Assurance. By Wm. Brinton, M.D. London: Churchill. 1856.


**Published:** 1856-07

**Authors:** 


### THE MEDICAL SELECTION OF LIVES FOR ASSURANCE.*

Dr. Brinton gives some useful and excellent rules on this important practical subject. Pulmonary tubercle is dwelt on as the most objectionable feature in a case proposed for insurance. When, in addition to a consumptive father or mother, the applicant for insurance has also a consumptive brother or sister, the life should be declined. "The reformed drunkard is not a good life." A man who practises a sedentary occupation in a dark room is a bad life. Men who take a great deal of fatigue and of unrest are bad lives. In considering the build of a person, weight should be taken into account together with height. If the height be 66 inches, the average weight should be 140 lbs. avoirdupois. Twenty per cent., or one-fifth, is the maximum variation from this rule. Rapid or sudden variations in weight are always objectionable as indicating doubtful health.

Dr. Brinton's special experience leads him also to think,

* The Medical Selection of Lives for Assurance. By Wm. Brinton, M.D. London: Churchill. 1856.

that streaks of red bloodvessels (in which the vessels are marked out) on a pale cheek, are a sign of the disease of the kidney, which gives rise to albuminuria. Persons suffering from open ulcers and sores are bad lives. The spirometer is of great service in some cases. A healthy male, of from 15 to 50 years old, averaging 10 stone in weight and 66 inches in height, should show a vital capacity of about 200 inches. For every inch of height above or below 66 inches, and within the ranges of 60 and 72, eight and a half cubic inches in capacity may be subtracted or added, from or to every inch below or above the usual standard of height.

An intermittent pulse is a doubtful sign, and should receive in all cases an explanation.

